# Efficacy of the Insecticide Formulation SumiShield^®^ 50WG for Malaria Vector Control in Experimental Huts in Madagascar

**DOI:** 10.4269/ajtmh.24-0811

**Published:** 2025-10-23

**Authors:** Thiery Nepomichene, Rico Randrenjarison, Jaritiana Randriamanga, Romain Girod

**Affiliations:** Medical Entomology Unit, Institut Pasteur de Madagascar, Antananarivo, Madagascar

## Abstract

Indoor residual insecticide spraying and the distribution of insecticide-treated nets have undoubtedly led to a significant reduction in the global malaria burden. However, insecticide resistance poses a threat to the effectiveness of these recommended control methods. In the present study, the aim was to determine the efficacy of SumiShield^®^ 50WG (Sumitomo Chemical Co. Ltd., Tokyo, Japan), an insecticide containing clothianidin, a neonicotinoid, in controlling malaria vectors in Madagascar. The study was conducted over 9 months after the initial spraying in experimental huts made with walls coated using different substrates, and both wild anopheline mosquitoes and an *Anopheles arabiensis* (*An. arabiensis*) insecticide-susceptible laboratory strain were used. Mortality in wild mosquitoes remained above the WHO threshold of 80.0% for up to 8 months post-spray, when assessed up to 96 hours after capture, depending on the type of wall surface. SumiShield 50WG did not induce exophily or inhibit blood-feeding in wild mosquitoes because no significant differences were observed between treated and control huts regarding the rates of exophily and blood-fed mosquitoes. In *An. arabiensis*, the WHO mortality threshold was also met for up to 8 months post-spray when assessed up to 96 hours after exposure. However, during the ninth month, this threshold was not achieved, even when mortality was assessed up to 120 hours after exposure. The residual efficacy of the formulation, which lasts up to 8 months, is sufficient to cover the malaria transmission season in most endemic areas of Madagascar.

## INTRODUCTION

Indoor residual spraying (IRS) and the distribution of insecticide-treated nets, along with larval control in specific situations, constitute the most effective interventions for malaria vector control. These interventions have contributed significantly to the substantial reduction in malaria morbidity and mortality currently observed in Sub-Saharan Africa.^1^ Unfortunately, anopheline mosquitoes have developed a resistance mechanism to some or most of the insecticides traditionally recommended for their control, and this phenomenon is considered the largest threat to the effectiveness of such interventions. Under these conditions, one of the primary operational recommendations is to alternate the use of insecticide classes, with the goal of limiting the development of resistance.^2^ Four insecticide classes were historically used for IRS, including organochlorines, organophosphates, carbamates, and pyrethroids. In 2017, another insecticide belonging to a fifth class, the neonicotinoid class, was used in IRS. Until mid-2024, ∼25 IRS products belonging to these five classes were prequalified by the WHO.^3^

Beyond the problem of insecticide resistance, some insecticide formulations used for IRS have a short residual life on local wall substrates^4^^–^^7^ and thus may require multiple rounds of application to cover extended malaria transmission seasons or in areas where perennial transmission occurs. However, IRS is logistically challenging to implement.^2^ Such features of IRS pose a major challenge to most of sub-Saharan Africa. Therefore, the identification and evaluation of insecticide formulations with a long insecticidal effect duration that do not exhibit cross-resistance to commonly used insecticides are vital.

In Madagascar, well-known malaria vector species include *Anopheles gambiae* (*An. gambiae*), *Anopheles arabiensis* (*An. arabiensis*), *Anopheles funestus* (*An. funestus*), *Anopheles mascarensis* (*An. mascarensis*), and *Anopheles merus* (*An. merus*).^8^^–^^11^ Another species, *Anopheles coustani* (*An. coustani*), was also described as a potential vector.^12^^,^^13^ In addition, the vectorial role of *Anopheles squamosus* (*An. squamosus*), another very abundant and zoo-anthropophilic species, is questionable. Intensive IRS campaigns designed to eliminate malaria vectors began in 1949 with the application of dichloro-diphenyl-trichloroethane (DDT) insecticide and were implemented successfully throughout the country until the 1970s, leading to the interruption of transmission in many parts of the Central Highlands and a significant decrease in morbidity in perennial transmission areas.^14^ Unfortunately, after control measures were abandoned during subsequent years, malaria progressively reemerged, with deadly outbreaks occurring during the 1980s.^15^ Indoor residual spraying with DDT was reintroduced in 1993; however, the resistance of *An. gambiae s.l.* to this insecticide was rapidly detected.^16^ In 2005, the Malagasy National Malaria Control Program changed its approach and replaced DDT with pyrethroids for IRS in the Central Highlands. After the discovery of the resistance of *An. gambiae* and *An. arabiensis* to various pyrethroids, carbamates were used from 2009 to 2013. In compliance with the adoption of a strategy involving the alternation of insecticides, organophosphates have been used since 2014. Recently, clothianidin was also introduced into the range of insecticides used for IRS in Madagascar. Nowadays, *An. gambiae* and *An. arabiensis* have been shown to be resistant to DDT and pyrethroids in some areas, whereas *An. mascarensis* is considered fully susceptible to insecticides of all families.^17^ *Anopheles funestus* has been shown to be resistant to pyrethroids and bendiocarb in a single village in the northwestern region.^18^ However, no resistance to clothianidin has been observed in Madagascar.^19^ Although insecticide resistance in *Anopheles* populations is not yet a major obstacle to malaria control in Madagascar, compared with other countries. Therefore, proactive measures to limit its future spread should be adopted as a priority.

Clothianidin is an insecticide that belongs to the Neonicotinoid family. This insecticide has been widely used in agriculture and as a veterinary ectoparasiticide, and it has recently been proposed for use in public health interventions. Clothianidin acts as an agonist of nicotinic acetylcholine receptors. This mode of action is different from those of organochlorines, organophosphates, carbamates, and pyrethroids. This compound is therefore valuable for controlling anopheline mosquitoes in the context of resistance emergence or in combination with commonly used insecticides. Additionally, because Neonicotinoids have a low affinity for vertebrate nicotinic receptors and do not bioaccumulate in the environment, these molecules are suitable insecticide candidates from an ecotoxicological perspective.^20^

SumiShield^®^ 50WG (Sumitomo Chemical Co. Ltd., Tokyo, Japan) is an insecticide formulation that contains clothianidin as its active ingredient. It was developed by Sumitomo Chemical Co. Ltd. for use in IRS to control malaria vectors. A study conducted in Benin has revealed that SumiShield 50WG is efficient against pyrethroid-resistant mosquitoes, leading to a mean mortality of 91.7% over an 8-month period and exceeding the WHO’s 80% mortality threshold for a susceptible strain for 32 weeks post-treatment.^21^ Another study conducted under experimental conditions has also revealed that clothianidin is effective against both susceptible and resistant *An. gambiae* populations for 18 months post-treatment.^22^ A separate study in Ethiopia revealed that the effectiveness of this insecticide lasts up to 9 months post-treatment.^23^

The primary objective for the present study was to determine the effectiveness and residual activity of SumiShield 50WG as an insecticide formulation for IRS programs in Madagascar. The specific objectives were to 1) evaluate the efficacy of SumiShield 50WG against natural malaria vector populations, examining immediate and delayed mortality, the inhibition of blood feeding, and induced exophily observed in mosquitoes, and 2) determine the persistence of the insecticidal or lethal action of the formulation against an insecticide-susceptible laboratory strain of *An. arabiensis* over time.

## MATERIALS AND METHODS

### Study site and period.

**Figure 1.**
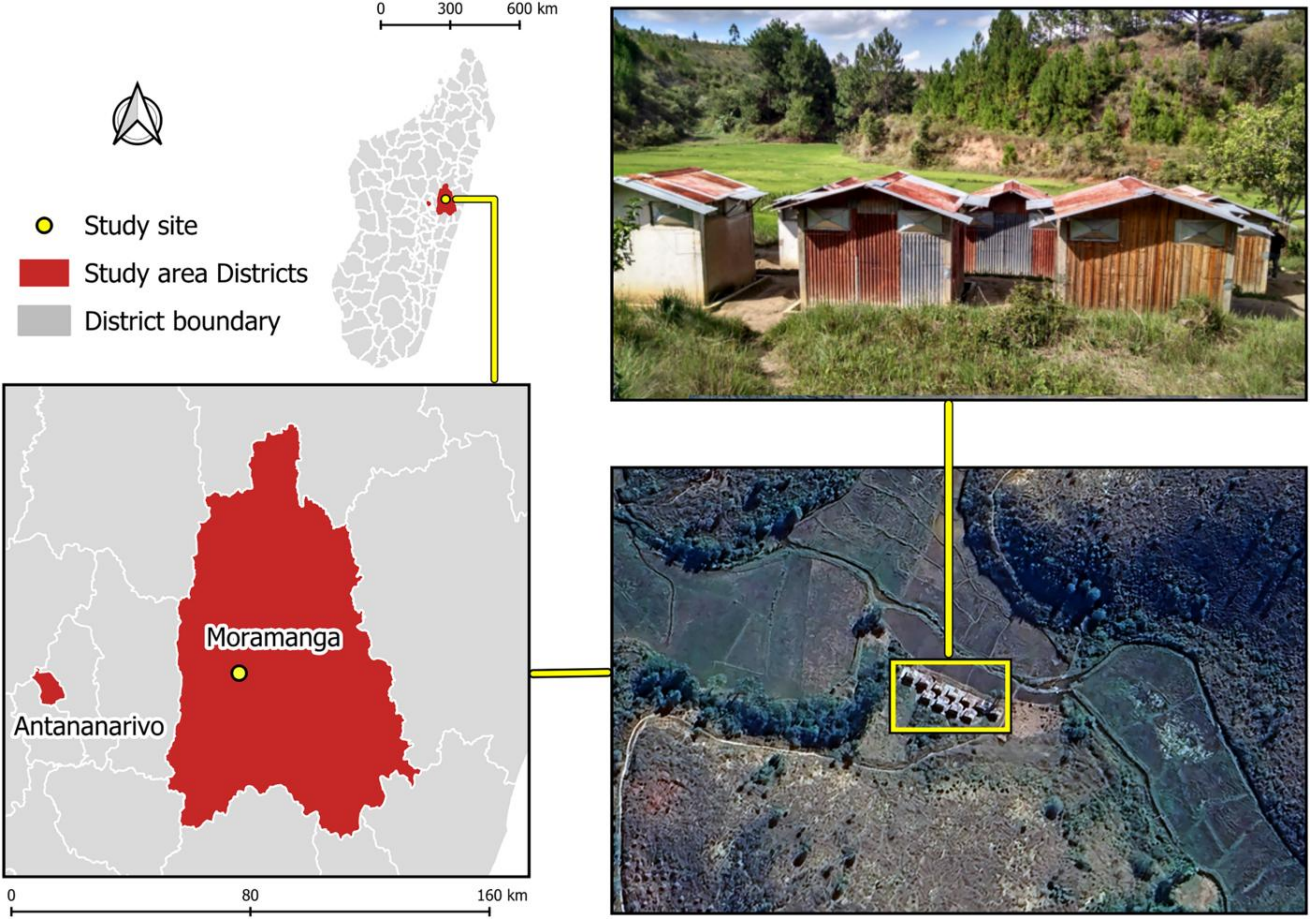
Study sites, located on the eastern edge of the Central Highlands of Madagascar, 100 km east of the capital Antananarivo, in the Moramanga District of the Alaotra-Mangoro Region.

The present study was conducted in the experimental hut station of the Institut Pasteur de Madagascar (IPM) of Saharevo (18°51′12.9″ S, 48°07′48.8″ E). This station is located on the eastern edge of the Central Highlands of Madagascar, 100 km east of the capital Antananarivo, in the Moramanga District of the Alaotra-Mangoro Region (Figure 1), where the climate is tropical. In this region, the hot and wet season lasts from October to April, whereas the cold and dry season lasts from May to September. This station is built near a village located in a rice-growing area, where *Anopheles* densities are heavily dependent on rice-planting cycles. Previous entomological investigations in Saharevo revealed the presence of 12 anopheline species, including four primary malaria vectors: *An. funestus*, *An. gambiae*, *An. arabiensis*, and *An. mascarensis*.^24^ All studies conducted at this station since then have confirmed the presence of these four species. *Anopheles coustani*, which is now considered a potential malaria vector in Madagascar, is also present in high densities. *Anopheles squamosus*, another abundant and zoo-anthropophilic species with questionable implications in malaria transmission, is also frequently collected.^24^^,^^25^

The vector control strategy adopted in the village of Saharevo and its surrounding villages before the present study was based on the distribution of long-lasting insecticide-treated bed nets. For several years, deltamethrin-impregnated nets were distributed. Focused IRS operations were conducted in some localities of the Moramanga District as needed. The insecticide used was typically pirimiphos-methyl.

Part of the present work was also conducted in the neighboring village of Ambohitranivo (18°50′ 52.7″ S, 48°14′ 24.2″ E), where additional mosquito collections were performed, as well as in the field insectary of the IPM, located in the town of Moramanga, ∼20 km from the field station of Saharevo, where mosquitoes can be reared under satisfactory conditions. Initial spraying was performed at the end of January 2019. Ten post-application mosquito collection sessions were then organized: 1 week after spraying, in early February, and every month, spanning the period from early March to early November 2019.

### Experimental hut description.

The experimental huts were designed in accordance with the WHO specifications for the standard evaluation of IRS.^26^ They were made of local materials generally used to build houses in Madagascar. A total of 11 huts were used: 1) two huts with walls made of mud (made by mixing of local clay and water), 2) two huts with walls made of cement (mixed with sand and water), 3) two huts with walls made of wood (pine planks), 4) two huts with walls made of tin (galvanized iron sheets), and 5) two huts with walls made of vegetal materials (the stems and leaves of local palm trees). Each type of hut was duplicated to ensure that for each type of wall substrate, a hut without insecticide could serve as a negative control. These control huts were used in conjunction with the huts that had insecticide-treated walls. An additional eleventh composite hut, composed of the different wall substrates mentioned above, was treated with another reference insecticide formulation and used as a positive control.

All huts were 3 meters long, 2 meters wide, and 2.5 meters high at the ridge. The floor was covered with a thin layer of cement. A plastic tarpaulin ceiling was stretched beneath the roof to act as a ceiling, facilitating the collection of mosquitoes. Each hut was surrounded by a gutter regularly filled with water to prevent ants, spiders, and other potential mosquito predators from entering. Mosquitoes were intended to enter the huts through four window slits made of an iron sheet, 1 cm wide, located on three sides of each hut (two on the side facing the rice fields and two on each of the two sides of the hut). Four window exit traps, each consisting of a mosquito net cage, were arranged similarly, allowing for the collection of mosquitoes that exited through the windows. The huts were located in the valley, situated just in front of the rice fields, more than 500 m from the village of Saharevo (Figure 1).

### Insecticide formulations and initial spraying.

Two insecticide formulations were used during the study: SumiShield 50WG and Actellic^®^ 300CS (Syngenta, Basel, Switzerland). A total of five huts, each built with a different material, were treated with SumiShield 50WG. Five other untreated huts served as negative controls. The eleventh composite hut was treated with Actellic 300CS. SumiShield 50WG is a formulation that involves water-dispersible granules containing clothianidin at a concentration of 500 g/kg. The intended dosage rate for treated surfaces was 300 mg/m^2^ of clothianidin. Actellic 300CS was used as a positive control. Actellic 300CS is a microencapsulated suspension of pirimiphos-methyl at a concentration of 300 g/L. The intended dosage rate on treated surfaces was 1,000 mg/m^2^ of pirimiphos-methyl. Initial spraying was performed by experts from Sumitomo Chemical Co., Ltd.

### Rainfall, temperature, and relative humidity data collection during the study period.

Temperature, precipitation, and relative humidity were recorded during each working session in the experimental station. Temperature and relative humidity were measured hourly using an Onset Hobo^®^ data logger (Onset Computer Corp., Bourne, MA) and Hoboware^®^ software (Onset Computer Corp.). The device was placed in the shade outside the huts. Cumulative precipitation was recorded daily using a rain gauge located near the experimental huts. The mean temperature and relative humidity, as well as the cumulative precipitation, were then evaluated during each work session.

### Local vector susceptibility testing to insecticide evaluation.

Local anopheline mosquitoes were tested according to WHO specifications to check their susceptibility to deltamethrin, pirimiphos-methyl, and clothianidin.^19^ These active ingredients were selected because they are used in the composition of insecticide formulations or impregnated materials used for malaria vector control in Madagascar. Additional formulations containing deltamethrin or clothianidin were also used in Madagascar. The susceptibility of mosquitoes to insecticides was tested by exposing females to papers impregnated with these active ingredients. Papers impregnated with deltamethrin and pirimiphos-methyl were provided by the WHO, with diagnostic doses of 0.05% and 0.25%, respectively. Papers impregnated with clothianidin were provided by Sumitomo Chemical Co. Ltd., with a proposed diagnostic dose of 2.00%. Wild female mosquitoes were collected in zebu parks in the villages of Saharevo and Ambohitranivo in February and March 2019. Briefly, females were collected using a mouth aspirator in the evening when resting in nonimpregnated nets that had been installed throughout parks containing two or more zebus. Engorged females were then brought back to the field insectary in Moramanga, reared until they laid eggs, and identified morphologically or molecularly (as described below). The first generation of mosquitoes from each species was then reared until they reached the adult stage. Females aged 3–5 days were used for tests conducted using WHO standard tubes. A total of 100 female mosquitoes were exposed to each active ingredient in WHO tubes,^27^ whereas 50 female mosquitoes served as negative controls. Exposure lasted 1 hour, after which the knockdown effect was recorded. Finally, mortality was recorded 24 hours after exposure to deltamethrin and pirimiphos-methyl and 48 hours after exposure to clothianidin. Any mosquito that could not stand or fly properly was considered dead.

According to WHO criteria, anopheline mosquito populations are considered susceptible to the active ingredient tested if their mortality rate exceeds 98%, tolerant if their mortality rate falls between 90% and 97%, and resistant if their mortality rate is below 90%.^27^

### Efficacy of SumiShield 50WG on wild anopheline mosquitoes.

The impact of SumiShield 50WG treatment on wild anopheline mosquitoes was studied 1 week after treatment (early February 2019) and then monthly for 9 months until November 2019. Each month, 10 successive nights of mosquito collection were implemented at the Saharevo station. Ten local adults, recruited as volunteers, slept on mats and collected mosquitoes in the five sprayed huts and the five control huts. A supervisor was also recruited to organize collections and collect mosquitoes in the traps outside the huts. The 10 volunteers changed huts each night, so that everyone eventually worked in each of the 10 huts during the 10 successive nights of the survey. The purpose of this design was to minimize bias caused by differences in the attractiveness of sleepers, mitigating any potential effects on the interpretation of the results.

No mosquito collection was performed in the composite hut during this part of the study. The day before the first night of collection, a dish of dead mosquitoes was placed on the floors of the huts. The dish was picked up before starting collection to ensure the absence of mosquito predators, such as ants or spiders. Each night of collection, at 6:30 pm, the supervisor opened the four window slits and the four window traps on each hut. To simulate the normal behavior of inhabitants, a candle was lit inside each hut. The 10 sleepers entered the huts at 7:30 pm and blew out the candle 15 minutes later. Subsequently, they slept from 8:00 pm to 5:30 am, at which time they closed the window traps. The entry slits were closed by the supervisor. Mosquitoes were then collected from the floor, walls, and ceiling by the mosquito collectors, while mosquitoes in the window traps were collected by the supervisor. Mosquito collections were performed using individual tubes, cotton, and torches. All mosquitoes were scored by location of collection and transported with a refrigerated cooler to the insectary in Moramanga. Mortality and engorgement status were also recorded. Mosquitoes were held in individual netted plastic cups and supplied with a 10% sugar solution during observation in the insectary. Mortality was recorded every 24 hours until 120 hours post-collection.

All mosquitoes caught were identified to the species level when possible. Anopheline mosquitoes from the *An. gambiae* complex were identified via polymerase chain reaction testing to discriminate among the sibling species *An. arabiensis*, *An. gambiae*, *Anopheles coluzzii*, and *An. merus*, according to Wilkins et al.^28^

The following outcomes were used to assess the efficacy of the treatment with SumiShield 50WG in the experimental huts: 1) immediate mortality corresponding to the percentage of dead mosquitoes on the morning of collection, 2) delayed mortality corresponding to the percentage of dead mosquitoes at 24 hours, 48 hours, 72 hours, 96 hours, and 120 hours post-collection, 3) blood feeding rate corresponding to the percentage of blood-fed females, and 4) exit rates corresponding to the percentage of mosquitoes collected from window traps.

### Residual efficacy of SumiShield 50WG on an insecticide-susceptible *Anopheles* strain.

The study of the residual efficacy of SumiShield 50WG on various wall substrates was conducted in conjunction with the study of the impact of the formulation on wild anopheline mosquitoes, 1 week after the initial spraying in February 2019 and then at monthly intervals from March to November 2019. Standard WHO cone tests were performed in each type of hut, including all treated and untreated huts at the experimental station.^26^ For each type of substrate, the untreated hut served as a negative control. The composite hut treated with Actellic 300CS was used as a positive control.

The residual efficacy of the insecticide was evaluated using mosquitoes from a strain of *An. arabiensis* that is susceptible to the insecticides commonly used for vector control, which has been maintained and checked for its susceptibility status for several years in the insectary of the IPM in Antananarivo.^29^ To this end, mosquitoes in larval stages were carefully transferred using a refrigerated cooler box to the insectary in Moramanga each month for field trials.

Each month, ∼40 mosquitoes were tested on each type of wall substrate, including surfaces treated with SumiShield 50WG or Actellic 300CS, as well as untreated surfaces. Approximately 10 3- to 5-day-old female mosquitoes of the *An. arabiensis* species were introduced into each of four cones randomly fixed to each of the four walls of both the treated and control huts. Ten 3- to 5-day-old females were also introduced into each of five cones randomly affixed to the five different wall surfaces in the composite hut where Actellic 300CS was sprayed. Mosquitoes were exposed to the wall surfaces for 30 minutes and then immediately transferred into individual netted plastic cups. The location of each cone and its associated resulting mortality was recorded to allow for an improved interpretation of the effect of cone position on mosquito mortality. Cone locations were marked to avoid using the same location twice (because cone taping and insect handling can remove insecticide residue from the hut walls). Mosquitoes were then immediately transported using a refrigerated cooler box to the insectary in Moramanga for observation, where they were provided with a 10% sugar solution. Mortality was recorded at 24 hours, 48 hours, 72 hours, 96 hours, and 120 hours post-exposure.

## STATISTICAL ANALYSES

The effect of SumiShield 50WG on wild anopheline mosquitoes throughout the present longitudinal study was analyzed by pooling data across species. This method was necessary because of the low numbers of anopheline mosquitoes collected, particularly the low densities of the major malaria vectors. When considering wall surfaces or periods of collection post-spraying, the proportions of dead females, blood-fed females, and exophilic females were compared between the treated and control huts using the χ^2^ test. According to WHO criteria, insecticide treatment is considered effective when the immediate or delayed mortality rate of wild anopheline mosquitoes is at least 80.0%.^26^

To determine the residual efficacy of SumiShield 50WG applied to various types of wall surfaces against the susceptible *An. arabiensis* strain throughout the study, mosquito mortality in treated and control huts was analyzed according to WHO criteria.^26^ The efficacy of the insecticide formulation was considered acceptable when mortality rates of exposed mosquitoes in treated huts exceeded 80% and those in corresponding control huts were less than 20%. Abbott’s correction was applied to determine the mortality rates in treated huts when the mortality rates in the corresponding control huts were greater than 5%. All statistical analyses were performed at a significance level of 5.0%, using R version 4.0.2 (R Foundation, Vienna, Austria).^30^

## RESULTS

### Rainfall and temperature data collection during the study period.

Cumulative rainfalls during each 10-day period of mosquito collection in the huts reached 21.7 mm in March. No rain was observed during field sessions conducted in February, May through July, or September. The mean temperature during the 10-day periods of mosquito collection in huts varied from 17.0°C in August to 25.0°C in November.

### Insecticide susceptibility testing of local anopheline mosquito populations.

WHO standard tube tests were conducted on *An. arabiensis* and *An. coustani* F1 females aged 3–5 days. These females were obtained from mixtures of wild mosquitoes collected in zebu parks in the villages of Saharevo and Ambohitranivo, Moramanga District, in January 2019. The number of knocked down and dead *An. arabiensis* and *An. coustani* exposed to deltamethrin, pirimiphos-methyl, and clothianidin are shown in Table 1. According to WHO criteria, *An. arabiensis* and *An. coustani* from the villages of Saharevo and Ambohitranivo, located close to the Saharevo IPM experimental station, were susceptible to deltamethrin (0.05%), pirimiphos-methyl (0.25%), and clothianidin (2.00%).

**Table 1:** Susceptibility to deltamethrin, pririmiphos-methyl, and clothianidin of *Anopheles arabiensis* and *Anopheles coustani* in the villages of Saherevo and Ambohitranivo, Moramanga District, January 2019

Species	Insecticide	Exposition	Nb Mosquitoes Exposed	Knocked Down Mosquitoes (1 hour [%])	Dead Mosquitoes (24 or 48 hours* [%])
*Anopheles coustani*	Deltamethrin 0.05%	Control	50	1 (2.0)	0 (0.0)
		Treated	98	78 (79.6)	98 (100.0)
	Pirimiphos-methyl 0.25%	Control	50	1 (2.0)	1 (2.0)
		Treated	99	95 (96.0)	99 (100.0)
	Clothianidin 2.00%	Control	50	1 (2.0)	1 (2.0)
		Treated	100	5 (5.0)	100 (100.0)
*Anopheles arabiensis*	Deltamethrin 0.05%	Control	50	1 (2.0)	1 (2.0)
		Treated	84	80 (95.2)	84 (100.0)
	Pirimiphos-methyl 0.25%	Control	45	1 (2.2)	2 (4.4)
		Treated	100	100 (100.0)	100 (100.0)
	Clothianidin 2.00%	Control	50	0 (0.0)	0 (0.0)
		Treated	100	7 (7.0)	100 (100.0)

Nb = number.

* Mortality was recorded 24 hours after exposure to deltamethrin and pirimiphos-methyl and 48 hours after exposure to clothianidin.

### Efficacy of SumiShield 50WG on wild anopheline mosquitoes.

#### Mosquito collection.

In the present study, a total of 2,570 mosquitoes were collected over 10 collection sessions conducted from February to November 2019, including at least 18 mosquito species belonging to six genera: *Aedeomyia* (*N* = 15), *Aedes* (*N* = 130), *Anopheles* (*N* = 1,581), *Coquillettidia* (*N* = 1), *Culex* (*N* = 747), and *Mansonia* (*N* = 96).

#### Anopheline mosquito collection.

A total of 1,581 anopheline mosquitoes belonging to at least eight species were collected during the study period. *Anopheles coustani* and *An. squamosus* were predominant, representing 48.6% and 41.2% of the anopheline mosquitoes collected, respectively. Other anopheline species, such as *Anopheles flavicosta*, *An. mascarensis, An. gambiae*, *An. arabiensis*, and *An. funestus*, including the major malaria vectors known to be present in the area, were present in much smaller densities (Table 2).

**Table 2:** Anopheline mosquitoes captured, presented by species by month and treatment, at the Institut Pasteur de Madagascar experimental station of Saharevo, Moramanga District, from February to November 2019

Post-Spray Periods (months)	Treatments	*Anopheles arabiensis*	*Anopheles coustani*	*Anopheles flavicosta*	*Anopheles funestus*	*Anopheles gambiae*	*Anopheles mascarensis*	*Anopheles* sp.	*Anopheles squamosus*	Total (%)
1 Week (Feb.)	Ctl	1	104	4	0	0	1	0	55	165 (10.4)
	Trt	2	103	5	1	0	0	0	84	195 (12.3)
1 Month (Mar.)	Ctl	0	96	8	0	3	2	0	87	196 (12.4)
	Trt	0	113	1	1	1	1	2	83	202 (12.8)
2 Months (Apr.)	Ctl	0	93	3	0	0	5	0	83	184 (11.6)
	Trt	0	95	4	0	0	1	0	115	215 (13.6)
3 Months (May)	Ctl	0	30	15	0	1	3	0	10	59 (3.7)
	Trt	0	34	3	0	0	2	0	9	48 (3.0)
4 Months (Jun.)	Ctl	0	1	1	0	0	0	0	0	2 (0.1)
	Trt	0	26	0	0	0	0	0	4	30 (1.9)
5 Months (Jul.)	Ctl	0	3	7	0	0	6	2	4	22 (1.4)
	Trt	0	12	7	0	2	5	1	11	38 (2.4)
6 Months (Aug.)	Ctl	0	8	1	0	3	1	0	11	24 (1.5)
	Trt	0	5	0	0	2	7	1	7	22 (1.4)
7 Months (Sep.)	Ctl	0	1	22	0	4	0	0	7	34 (2.2)
	Trt	0	14	3	0	1	0	0	33	51 (3.2)
8 Months (Oct.)	Ctl	0	2	2	0	2	1	1	9	17 (1.1)
	Trt	0	3	5	0	0	0	0	11	19 (1.2)
9 Months (Nov.)	Ctl	0	13	0	0	0	0	0	16	29 (1.8)
	Trt	4	13	0	0	0	0	0	12	29 (1.8)
Total (%)		7 (0.4)	769 (48.6)	91 (5.8)	2 (0.1)	19 (1.2)	35 (2.2)	7 (0.4)	651 (41.2)	1,581 (100.0)

Ctl = negative control huts; Trt = SumiShield^®^ 50WG-treated huts. *Anopheles coustani* was the most abundant species captured (48.6% of anopheline mosquitoes), followed by *An. squamosus* (41.2% of anopheline mosquitoes).

Among 1,581 anopheline mosquitoes collected during the present study, the number of individuals captured varied by hut type, ranging from 248 in vegetal huts to 412 in tin huts. The proportion of mosquitoes collected in tin huts was significantly higher than that observed in vegetal, wood, and cement huts (*P* <0.05). Similarly, the proportion collected in mud huts was significantly higher than that collected in vegetal and wood huts (*P* <0.05). No significant differences were found among the other hut types (Table 3).

**Table 3:** Total number of anopheline mosquitoes captured per wall type in Saharevo experimental huts in 2019, presented by month and treatment

Post-Spraying Periods (months)	Cement*		Mud^†^		Tin^†^		Vegetal^‡^		Wood*		Total (%)
	Ctr	Trt	Ctr	Trt	Ctr	Trt	Ctr	Trt	Ctr	Trt	
1 Week (Feb.)	24	36	32	69	51	46	30	18	28	26	360 (22.8)^§^
1 Month (Mar.)	48	38	39	41	38	59	38	34	33	30	398 (25.2)^§^
2 Months (Apr.)	40	29	40	43	32	87	34	31	38	25	399 (25.2)^§^
3 Months (May)	14	9	11	14	13	12	7	5	14	8	107 (6.8)^‖^
4 Months (Jun.)	1	6	0	8	1	7	0	4	0	5	32 (2.0)^¶^
5 Months (Jul.)	4	5	4	7	6	10	5	6	3	10	60 (3,8)^#^
6 Months (Aug.)	1	3	7	1	5	9	5	9	6	0	46 (2.9)**
7 Months (Sep.)	5	6	5	3	2	4	0	5	5	1	36 (2.3)^††^
8 Months (Oct.)	10	11	9	9	8	9	4	6	3	16	85 (5.4)**
9 Months (Nov.)	7	2	3	10	10	3	5	2	4	12	58 (3.7)**
	154	145	150	205	166	246	128	120	134	133	1,581 (100.0)

Ctl = negative control huts; Trt = SumiShield^®^ 50WG-treated huts.

*^, †, ‡, §, ‖, ¶, #, **, ††^ Symbol groups represent the results of pairwise χ^2^ tests (α = 0.05). Wall types and post-spraying periods that share the same symbol are not significantly different.

A total of 732 (46.3%) anopheline mosquitoes were collected in the control huts, and 849 (53.7%) were collected in treated huts. This difference was statistically significant (*P* <0.05). A significantly higher proportion of anopheline mosquitoes was collected in treated huts compared with control huts constructed with walls made of tin and mud (*P* <0.05). In contrast, no significant differences were observed between the treated and control huts made of cement, vegetal materials, or wood (*P* >0.05). With respect to temporal variation, a significant increase in mosquito collections in treated huts was observed only in June (*P* >0.05), whereas in all other months, the differences between treatments were not statistically significant (*P* <0.05). The overall mosquito density peaked during the first 3 months post-treatment (February to April), followed by a marked decline in subsequent months. Independent of treatment status, tin huts exhibited higher attractiveness to anopheline mosquitoes, whereas vegetal and wood huts appeared to be less attractive (Table 3).

#### Immediate and delayed mortality in anopheline mosquitoes.

The immediate and delayed mortality rates of anopheline mosquitoes were studied on the basis of post-spraying periods and the different types of huts. The immediate and delayed mortality rates of anopheline mosquitoes in all types of control and treated huts, grouped by collection session, are shown in Table 4. Immediate and delayed mortality rates observed in treated huts were significantly higher than those observed in corresponding control huts. With the exception of the tin hut, the WHO threshold was not reached in any of the huts, considering both immediate mortality and mortality after 24 hours. However, it was reached in cement, mud, and vegetal huts when considering mortality after 48 hours, as well as in wood huts when considering mortality after 72 hours.

**Table 4:** Immediate and delayed mortality of anopheline mosquitoes in the five types of control and treated huts, calculated by grouping all collection sessions, at the Institut Pasteur de Madagascar experimental station of Saharevo, Moramanga District, from February to November 2019

Hut Treatments	Wall Types	Total *Anopheles* Caught	Mortality (%)					
			Immediate	After 24 hours	After 48 hours	After 72 hours	After 96 hours	After 120 hours
Negative control huts	Cement	154	5.2	9.7	14.9	19.5	22.7	31.8
	Mud	150	6.0	12.7	18.7	22.0	30.7	32.0
	Tin	166	5.4	12.7	14.5	17.5	22.3	29.5
	Vegetal	128	11.7	18.0	18.8	20.3	21.1	27.3
	Wood	134	5.2	17.2	22.4	23.9	24.6	30.6
SumiShield^®^ 50WG-treated huts	Cement	145	32.4*	71.0*	**82.8** *	**89.7** *	**95.2** *	**96.6** *
	Mud	205	39.0*	76.6*	**88.3** *	**92.2** *	**94.6** *	**95.6** *
	Tin	246	50.4*	**82.5** *	**88.6** *	**92.3** *	**98.0** *	**98.4** *
	Vegetal	120	48.3*	75.8*	**80.8** *	**90.8** *	**94.2** *	**98.3** *
	Wood	133	21.8*	59.4*	76.7*	**85.7** *	**94.0** *	**94.0** *

The immediate and delayed mortality rates observed in treated huts were all significantly higher than those observed in the corresponding control huts. Bold type indicates values that exceed the WHO threshold.

* The mortality rate in the treated hut was significantly higher than that in the corresponding control hut.

The immediate and delayed mortality of anopheline mosquitoes, as observed during the 10 collection sessions, is shown in Table 5, grouped by the types of control and treated huts. Between 24 and 120 hours post-collection, significant differences in mosquito mortality were consistently observed between treated and control huts from 1 week to 8 months after spraying. During this period, the WHO threshold of ≥80% mortality was consistently reached in treated huts, confirming sustained insecticidal efficacy. At 9 months post-spraying, no significant differences in mortality were observed between treated and control huts at any time point, and the WHO threshold of ≥80% mortality was no longer reached in treated huts, indicating a complete loss of efficacy (Table 5).

**Table 5:** Immediate and delayed mortality of anopheline mosquitoes during the 10 collection sessions, calculated by grouping all types of control and treated huts, at the Institut Pasteur de Madagascar experimental station of Saharevo, Moramanga District, from February to November 2019

Hut Treatments	Post-Spraying Periods	Total *Anopheles* Caught	% Mortality					
			Immediate	After 24 hours	After 48 hours	After 72 hours	After 96 hours	After 120 hours
Negative control huts	1 Week	165	1.2	16.4	17.6	18.8	20.6	27.3
	1 Month	196	12.2	14.8	14.8	17.3	17.3	22.4
	2 Months	184	9.2	9.2	16.3	16.8	20.7	28.3
	3 Months	59	1.7	32.2	40.7	50.8	72.9	84.7
	4 Months	2	0.0	0.0	50.0	50.0	50.0	50.0
	5 Months	22	0.0	4.5	18.2	18.2	18.2	27.3
	6 Months	24	4.2	12.5	12.5	16.7	37.5	29.2
	7 Months	34	2.9	5.9	11.8	23.5	23.5	26.5
	8 Months	17	0.0	5.9	17.6	17.6	17.6	23.5
	9 Months	29	6.9	6.9	6.9	13.8	13.8	13.8
SumiShield^®^ 50WG-treated huts	1 Week	195	47.7*	**87.7** *	**96.9** *	**97.9** *	**98.5** *	**98.5** *
	1 Month	202	52.0*	**86.1** *	**94.6** *	**99.0** *	**100.0** *	**100.0** *
	2 Months	215	51.6*	**80.9** *	**92.6** *	**96.7** *	**100.0** *	**100.0** *
	3 Months	48	10.4*	**87.5** *	**100.0** *	**100.0** *	**100.0** *	**100.0**
	4 Months	30	16.7*	76.7*	**80.0**	**90.0** *	**96.7** *	**96.7**
	5 Months	38	0.0*	26.3*	47.4*	68.4*	**100.0** *	**100.0** *
	6 Months	22	45.5*	77.3*	77.3*	**81.8** *	**81.8** *	**95.5** *
	7 Months	51	9.8	29.4*	43.1*	60.8*	**82.4** *	**92.2** *
	8 Months	19	10.5	15.8	21.1	47.4	**84.2** *	**94.7** *
	9 Months	29	6.9	13.8	20.7	37.9	37.9	37.9

Bold type indicates values that exceed the WHO threshold.

* The mortality rate in the treated hut was significantly higher than that observed in the corresponding control hut.

#### Induced exophily and engorgement inhibition in anopheline mosquitoes.

Induced exophily and engorgement inhibition were studied according to post-spraying periods and the different types of huts. The percentage of anopheline mosquitoes collected in window traps and the percentage of anopheline mosquitoes engorged in the five types of control and treated huts, grouped by all collection sessions, are shown in Table 6. Exophily rates of anopheline mosquitoes were not significantly different among the various types of control and treated huts during the 10 collection sessions, except for tin and vegetal huts, where exophily rates were significantly lower in treated huts. The engorgement rates of anopheline mosquitoes were not significantly different among the various types of control and treated huts during the 10 collection sessions, except for vegetal huts, where the engorgement rate was significantly higher in treated huts.

**Table 6:** Proportion of engorged anopheline mosquitoes collected in window traps in the five types of control and treated huts, calculated by grouping all the collection sessions, at the Institut Pasteur de Madagascar experimental station of Saharevo, Moramanga District, from February to November 2019

Hut Treatments	Wall Type	Total *Anopheles* Caught	% Exophilic Mosquitoes	% Blood-Fed Mosquitoes
Negative control huts	Cement	154	34.4	2.6
	Mud	150	36.0	2.7
	Tin	166	36.1	3.0
	Vegetal	128	50.8	0.8
	Wood	134	32.1	3.7
SumiShield^®^ 50WG-treated huts	Cement	145	32.4	5.5
	Mud	205	26.8	4.9
	Tin	246	23.6*	4.5
	Vegetal	120	25.8*	3.3*
	Wood	133	25.6	5.3

* The percentage of exophilic and engorged anopheline mosquitoes in the treated hut was significantly different than that observed in the corresponding control hut.

Induced exophily and engorgement inhibition, as observed in the 10 collection sessions, are shown in Table 7. The exophily rates of anopheline mosquitoes were not significantly different between control huts and treated huts throughout the 10 collection sessions, except for the first month post-spraying (March 2019) and the second month post-spraying (April 2019), when exophily rates were significantly lower in treated huts. The engorgement rates of anopheline mosquitoes were not significantly different between control huts and treated huts over the 10 collection sessions, except for the first month post-spraying (March 2019), when engorgement rates were significantly higher in treated huts. These results suggest that SumiShield 50WG did not induce any exophilic behavior or engorgement inhibition in anopheline mosquitoes that entered the huts.

**Table 7:** Anopheline mosquito exophily rates and engorgement rates during 10 collection sessions, calculated by grouping all types of control and treated huts, at the Institut Pasteur de Madagascar experimental station of Saharevo, Moramanga District, from February to November 2019

Hut Treatments	Post-Spraying Periods	Total *Anopheles* Caught	% Exophilic Mosquitoes	% Blood-Fed Mosquitoes
Negative control huts	1 Week	165	47.3	3.0
	1 Month	196	27.0	2.6
	2 Months	184	41.3	4.9
	3 Months	59	33.9	0.0
	4 Months	2	100.0	0.0
	5 Months	22	36.4	0.0
	6 Months	24	45.8	0.0
	7 Months	34	35.3	0.0
	8 Months	17	23.5	0.0
	9 Months	29	37.9	0.0
SumiShield^®^ 50WG-treated huts	1 Week	195	38.5	4.1
	1 Month	202	10.9*	11.4*
	2 Months	215	17.7*	3.7
	3 Months	48	33.3	2.1
	4 Months	30	26.7	0.0
	5 Months	38	34.2	0.0
	6 Months	22	45.5	0.0
	7 Months	51	41.2	0.0
	8 Months	19	42.1	0.0
	9 Months	29	48.3	0.0

* The percentage of exophilic and engorged anopheline mosquitoes in the treated hut was significantly different than that observed in the corresponding control hut.

#### Residual efficacy of SumiShield 50WG on an insecticide-susceptible *Anopheles* strain.

The results of cone tests conducted throughout the 10-month study on walls treated with SumiShield 50WG and Actellic 300CS, as well as untreated walls, are presented for each type of wall surface as follows:

#### Cement huts.

**Figure 2.**
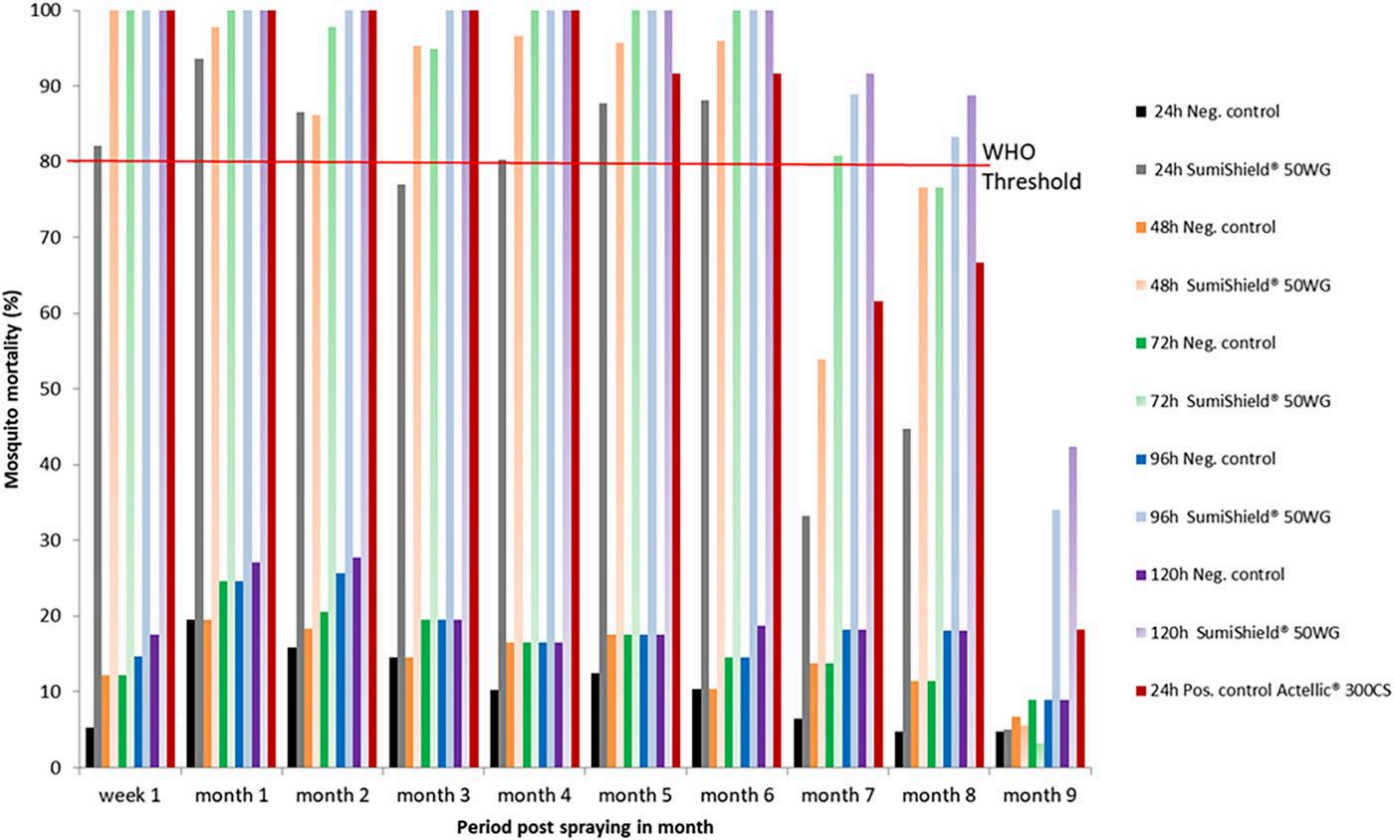
Mortality of insecticide-susceptible *Anopheles arabiensis* exposed to SumiShield^®^ 50WG and Actellic^®^ 300CS applied on walls made of cement at the Institut Pasteur de Madagascar experimental station of Saharevo, Moramanga District, from February to November 2019.

The residual efficacy of SumiShield 50WG treatment on cement walls is presented in Figure 2. Until the eighth month post-spraying, the mortality rate recorded exceeded the WHO threshold (>80%). The WHO threshold was reached after 24 hours post-exposure and continued until the sixth month post-spraying. However, during the seventh and eighth months, a mortality rate of 80% was reached at 72 hours and 96 hours post-exposure, respectively. During the ninth month post-spraying, mortality induced by SumiShield 50WG was below the WHO threshold, even at 120 hours post-exposure. Therefore, in the cement substrate, the bio-efficacy of SumiShield 50WG was observed until the eighth month post-spraying. The results for Actellic 300CS revealed a mortality rate greater than 80% at 24 hours post-exposure, which remained high, persisting until the sixth month post-spraying on walls made of cement.

#### Mud huts.

**Figure 3.**
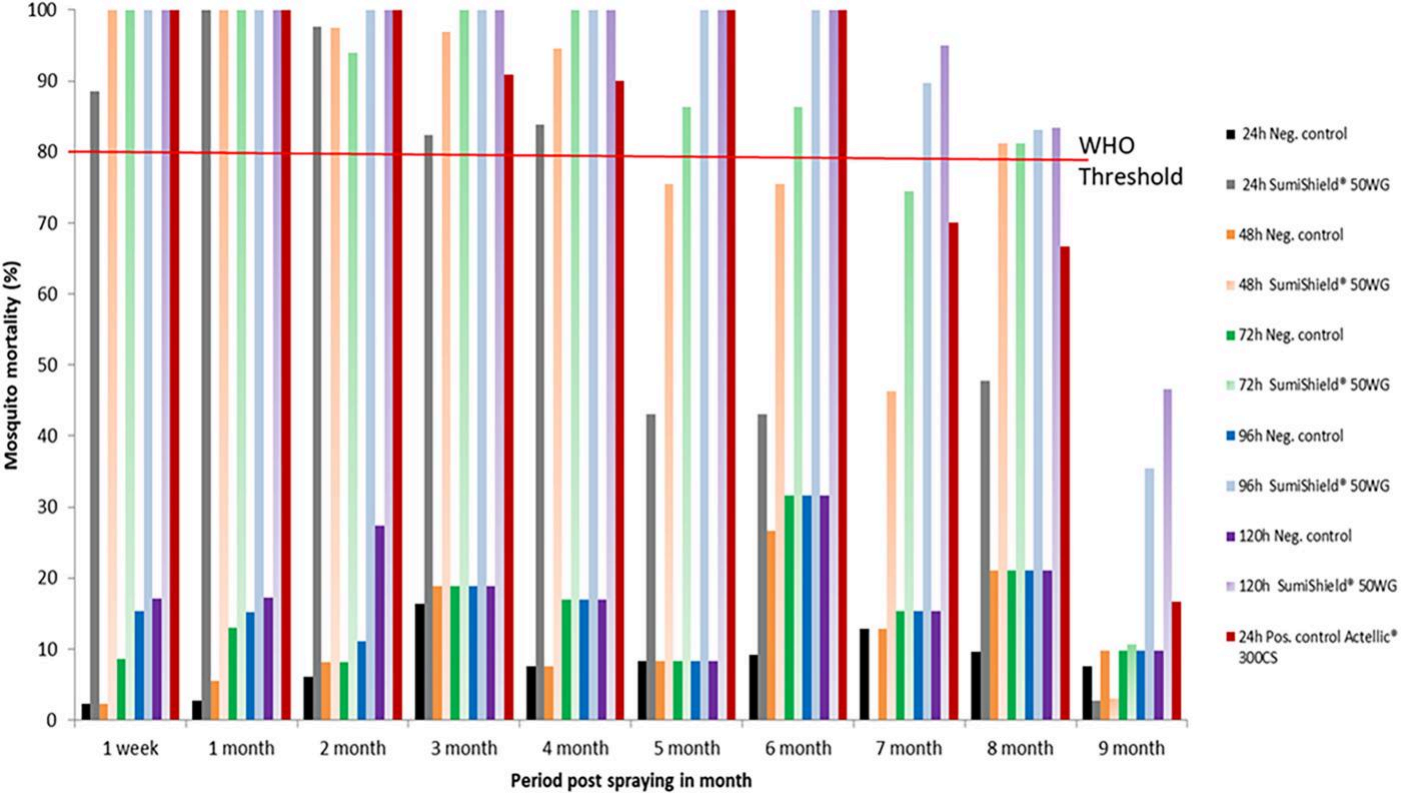
Mortality of insecticide-susceptible *Anopheles arabiensis* exposed to SumiShield^®^ 50WG and Actellic^®^ 300CS applied on walls made of mud at the Institut Pasteur de Madagascar experimental station of Saharevo, Moramanga District, from February to November 2019.

The residual efficacy of SumiShield 50WG treatment on mud walls is presented in Figure 3. Until the eighth month post-spraying, the mortality rate recorded exceeded the WHO threshold (>80%). The WHO threshold was reached at 24 hours post-exposure and remained stable until the fourth month post-spraying. However, during the fifth and sixth months, a mortality rate of 80% was reached at 72 hours post-exposure. During the seventh month, the threshold was reached at 96 hours post-exposure, and during the eighth month, it was reached at 48 hours. During the ninth month post-spraying, mortality induced by SumiShield 50WG fell below the WHO threshold, even at 120 hours post-exposure. Therefore, in the mud substrate, the bio-efficacy of SumiShield 50WG was also observed until the eighth month post-spraying. The results for Actellic 300CS also revealed a mortality rate greater than 80% after 24 hours post-exposure, persisting until the sixth month post-spraying on walls made of mud.

#### Tin huts.

**Figure 4.**
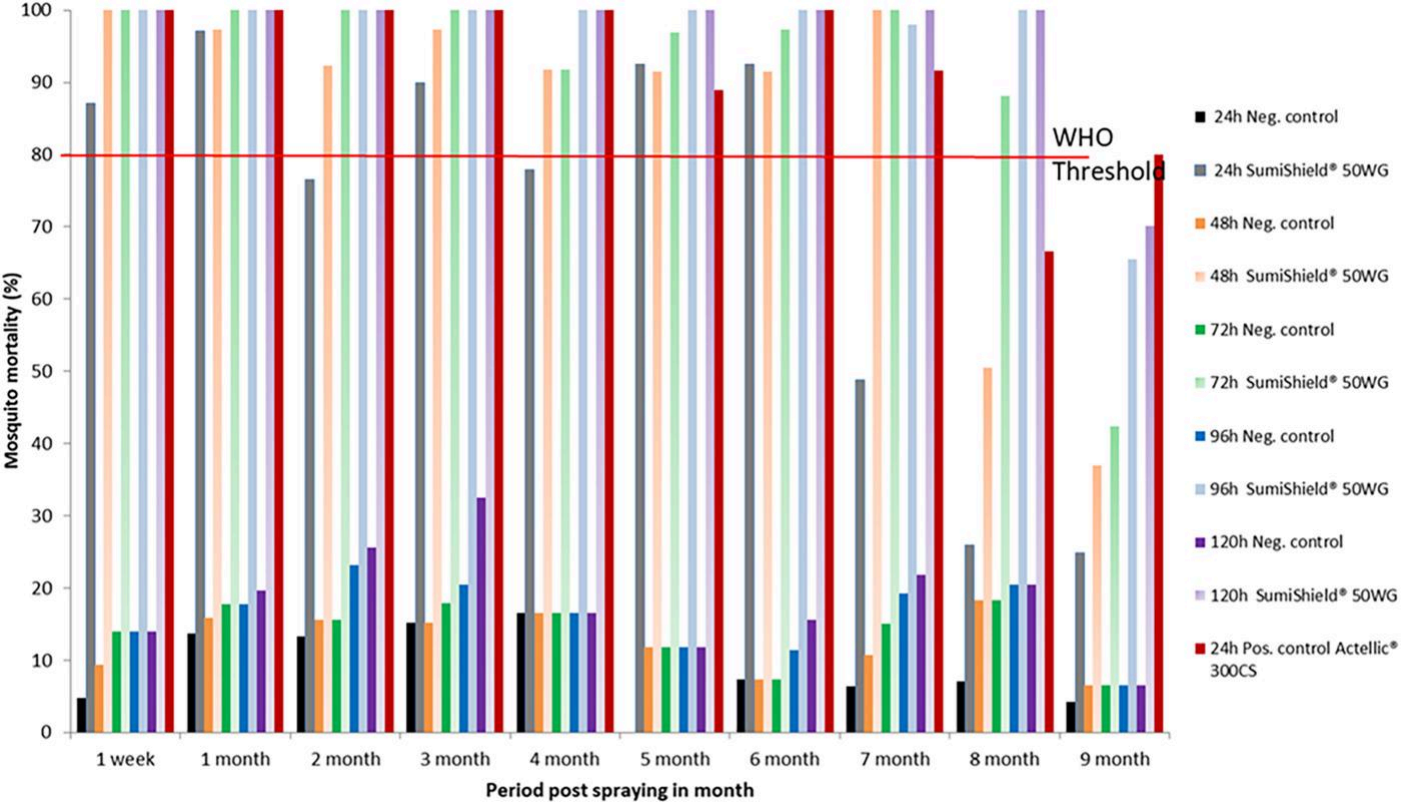
Mortality of insecticide-susceptible *Anopheles arabiensis* exposed to SumiShield^®^ 50WG and Actellic^®^ 300CS applied on walls made of tin at the Institut Pasteur de Madagascar experimental station of Saharevo, Moramanga District, from February to November 2019.

The residual efficacy of SumiShield 50WG treatment on tin walls is presented in Figure 4. Until the eighth month post-spraying, the recorded mortality rate exceeded the WHO threshold (>80%). The WHO threshold was reached at 24 hours post-exposure during the first week, first month, third month, fifth month, and sixth month post-spraying. In contrast, during the second, fourth, and seventh months post-spraying, a mortality rate of 80% was reached at 48 hours post-exposure. During the eighth month post-spraying, the threshold was reached at 72 hours post-exposure. During the ninth month post-spraying, mortality induced by SumiShield 50WG fell below the WHO threshold, even at 120 hours post-exposure. Therefore, in the tin substrate, the bio-efficacy of SumiShield 50WG was also observed until the eighth month post-spraying. The results for Actellic 300CS revealed a mortality rate greater than 80% at 24 hours post-exposure, persisting until the seventh month post-spraying on walls made of tin.

#### Vegetal huts.

**Figure 5.**
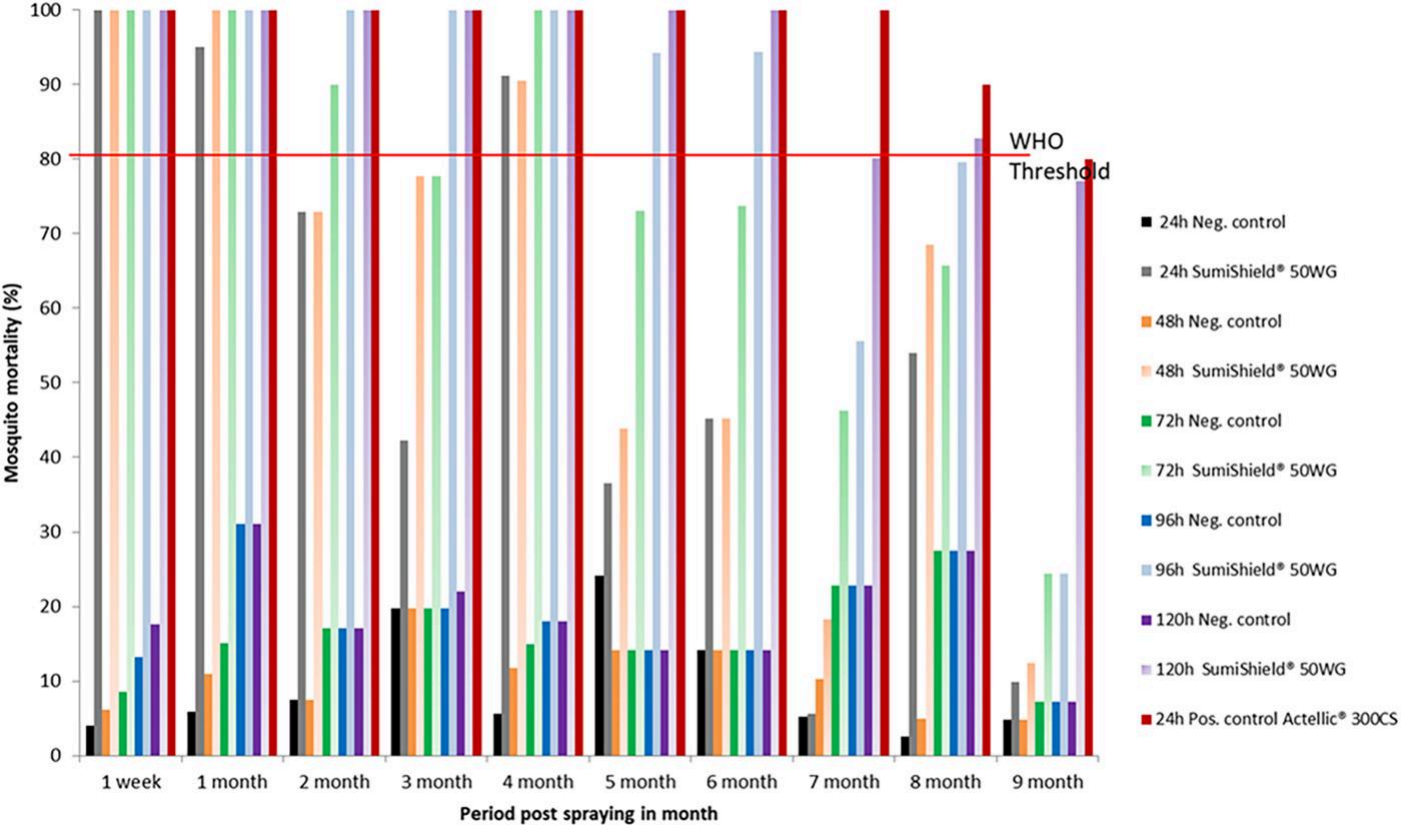
Mortality of insecticide-susceptible *Anopheles arabiensis* exposed to SumiShield^®^ 50WG and Actellic^®^ 300CS applied on vegetal walls at the Institut Pasteur de Madagascar experimental station of Saharevo, Moramanga District, from February to November 2019.

The residual efficacy of SumiShield 50WG treatment on vegetal walls is presented in Figure 5. Until the eighth month post-spraying, the recorded mortality rate exceeded the WHO threshold (>80%). The WHO threshold was reached at 24 hours post-exposure during the first week, first month, and fourth month post-spraying, whereas during the second month, a mortality rate of 80% was reached at 72 hours post-exposure. Additionally, a mortality rate of 80% was reached at 96 hours post-exposure during the third, fifth, and sixth months post-spraying. The WHO threshold was also reached at 120 hours post-exposure during the seventh and eighth months post-spraying. During the ninth month post-spraying, mortality induced by SumiShield 50WG fell below the WHO threshold, even at 120 hours post-exposure. Therefore, in vegetal substrate, the bio-efficacy of SumiShield 50WG was also observed until the eighth month post-spraying. The results for Actellic 300CS also revealed a mortality rate greater than 80%, persisting until the eighth month post-spraying at 24 hours post-exposure on vegetal walls.

#### Wood huts.

**Figure 6.**
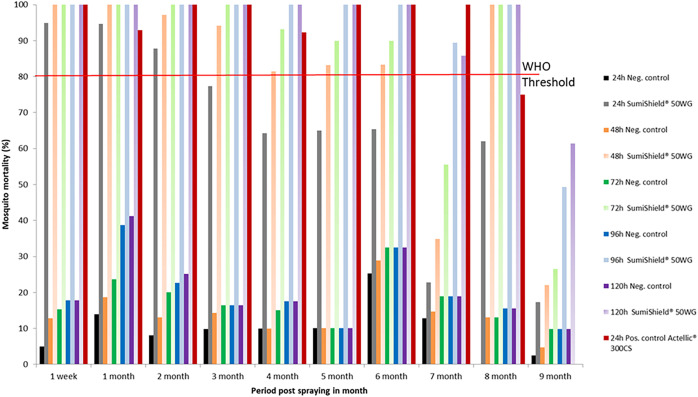
Mortality of insecticide-susceptible *Anopheles arabiensis* exposed to SumiShield^®^ 50WG and Actellic^®^ 300CS applied on walls made of wood at the Institut Pasteur de Madagascar experimental station of Saharevo, Moramanga District, from February to November 2019.

The residual efficacy of SumiShield 50WG treatment on wood walls is presented in Figure 6. Until the eighth month post-spraying, the recorded mortality rate exceeded the WHO threshold (>80%). The WHO threshold was reached at 24 hours post-exposure until the during month post-spraying, whereas from the third to sixth months post-spraying, as well as during the eighth month post-spraying, a mortality rate of 80% was reached at 48 hours post-exposure. During the seventh month post-spraying, a mortality rate of 80% was reached at 96 hours post-exposure. During the ninth month post-spraying, mortality induced by SumiShield 50WG fell below the WHO threshold, even at 120 hours post-exposure. Therefore, in the wood substrate, the bio-efficacy of SumiShield 50WG was observed until the eighth month post-spraying. The results of Actellic 300CS also revealed a mortality rate greater than 80%, persisting until the seventh month post-spraying at 24 hours post-exposure on walls made of wood.

## DISCUSSION and CONCLUSION

The bio-efficacy of the insecticide formulation SumiShield 50WG was evaluated under natural conditions in experimental huts made from substrates commonly used in Madagascar for house construction. The results suggest that SumiShield 50WG has a delayed toxic effect on wild anopheline mosquitoes in all five types of huts at the experimental station. An acceptable toxic effect was obtained after 24 hours, 48 hours, or 72 hours of observation, depending on the type of hut. No acceptable immediate mortality was observed, regardless of the type of hut. SumiShield 50WG exhibited a faster toxic effect on anopheline mosquitoes in tin huts, representing nonporous substrates, and a slower toxic effect in wood huts, representing porous substrates.

The results also reveal that SumiShield 50WG has a toxic effect on wild anopheline mosquitoes up to 8 months after the initial spraying of the huts at the experimental station. During the eighth month after spraying, the toxic efficacy was only observed for up to 96 hours after contact with the insecticide by the mosquitoes. During the seventh month after spraying, the toxic efficacy was observed for 24 hours after contact with the insecticide by the mosquitoes. During the 6 months after the initial spraying, SumiShield 50WG exhibited an immediate toxic effect, resulting in the immediate mortality of wild anopheline mosquitoes entering the huts.

Pyrethroid resistance is now widespread in malaria vector populations throughout sub-Saharan Africa and is emerging in Madagascar.^17^^,^^18^ Preliminary studies conducted by the manufacturer on SumiShield 50WG efficacy revealed promising efficacy against malaria vectors that lasted for several months in experimental hut trials in Africa. These studies could contribute to addressing the shortcomings of vector control strategies resulting from insecticide resistance.^31^ Results obtained through independent studies have reinforced these observations.^21^^,^^32^^,^^33^ Although the present study did not reveal post-exposure inhibition of blood-feeding, this effect has been previously reported with SumiShield 50WG under different conditions. As described by the manufacturer, although exposed mosquitoes are still alive, they are unable to bite or fly properly.^31^ This statement is important because SumiShield 50WG exhibits a delayed toxic effect. The absence of knockdown could increase exposure time to insecticide and consequently increase the quantity of insecticide absorbed through the cuticle of the mosquito, leading to death and preventing the development of resistance.

Moreover, anopheline mosquitoes were collected in similar numbers in the huts, regardless of the type of material used for their construction. A significant but unexplained difference was highlighted between tin and cement huts and wood and vegetal huts: the latter two appeared to be less attractive to anopheline mosquitoes. Regardless, the treated huts attracted no fewer anopheline mosquitoes than the control huts.

The total number of wild anopheline mosquitoes collected during the present longitudinal study, including primary and secondary malaria vectors, was adequate to assess immediate and delayed mortality, engorgement inhibition, and induced exophily; thus, the efficacy of the insecticide formulation initially sprayed on the walls of the huts of the experimental station was assessed. Nevertheless, because of the diversity of anopheline species, particularly the low densities of major malaria vectors collected throughout the study, data were pooled across anopheline species for analysis.

The immediate and delayed mortality of anopheline mosquitoes was analyzed by grouping all types of huts or all sessions together. The first interesting finding is that immediate and delayed mortality rates were significantly higher in treated huts, regardless of the type of hut. A notable finding is that the WHO threshold was not reached in any hut, considering both immediate mortality and mortality after 24 hours, with the exception of tin huts. However, it was reached in cement, mud, and vegetal huts when considering mortality after 48 hours and in wood huts when considering mortality after 72 hours. These results suggest that SumiShield 50WG exhibited a faster toxic effect on anopheline mosquitoes in tin huts, representing nonporous substrates, and a slower toxic effect on wood huts, representing porous substrates.

The second point constitutes the primary result of the longitudinal study. Except for rare exceptions, immediate and delayed mortality rates were significantly higher in treated huts during the first 6 months after spraying. During the seventh month post-spraying, significantly higher mortality rates were observed in treated huts 24 hours after exposure, but not immediately. During the eighth month post-spraying, significantly higher mortality rates were observed only after 96 hours had passed. During the ninth month post-spraying, no significant difference in mortality rates was observed between treated and control huts, even 120 hours after contact between anopheline mosquitoes and insecticide. A limiting point is that the WHO threshold was not reached, regardless of the collection session post-spraying, when considering immediate mortality. It was reached during the first 3 months post-spraying, when considering mortality after 24 hours, during the fourth month post-spraying, when considering mortality after 48 hours, and during the fifth through eighth months post-spraying, when considering mortality after 72 to 96 hours. The WHO threshold was never reached in treated huts during the ninth month after spraying. These results reveal that SumiShield 50WG has a significant effect on wild anopheline mosquito mortality up to 8 months post-spraying, with a significant immediate toxic effect observed for 6 months and a significant delayed toxic effect observed during the seventh and eighth months post-spraying.

The exophily and blood-feeding rates of anopheline mosquitoes were analyzed by grouping all types of huts or all sessions together. A significant difference in exophily rates was observed between treated and control huts for tin and vegetal huts only. However, this finding is counterintuitive because exophily rates were significantly lower in treated huts. Similarly, a significant difference in exophily rates was observed between treated and control huts 1 month and 2 months post-spraying. Again, exophily rates were curiously significantly lower in treated huts. These results suggest that SumiShield 50WG did not induce any exophilic behavior in anopheline mosquitoes that entered the huts.

A significant difference in blood feeding was observed between treated and control huts only for vegetal huts. Notably, the blood feeding rate was significantly higher in the treated hut. A significant difference in blood feeding was also observed between treated and control huts 1 month post-spraying. Again, the blood feeding rate remained significantly higher in the treated hut. These results suggest that SumiShield 50WG did not inhibit the engorgement of anopheline mosquitoes that entered the huts.

Insecticide-susceptible *An. arabiensis* were exposed to the different wall surfaces of the 11 huts at the IPM experimental station of Saharevo monthly from February to November 2019 to assess the residual efficacy of SumiShield 50WG. The primary result of the present longitudinal study was that the mortality rates observed in the treated huts remained above the WHO threshold until the eighth month post-spraying, regardless of the wall surface. During the ninth month post-spraying, mortality induced by SumiShield 50WG fell below the WHO threshold in all treated huts.

The WHO threshold was reached after longer observation times over the months and varied according to the type of hut. Notably, in tin and cement-treated huts, a mortality rate greater than 80% was observed only after 24 hours until the sixth month post-spraying. In contrast, the other huts required a delay of 48 hours (wood hut), 72 hours (mud hut), or 96 hours (vegetal hut) after insecticide exposure to reach a mortality rate greater than 80%. This mortality rate was reached from the third month post-spraying (in the wood hut) to the fifth month post-spraying (in the mud and vegetal huts). These results suggest that throughout the post-spraying period, SumiShield 50WG exhibited a faster toxic effect on anopheline mosquitoes when applied to nonporous substrates and a slower toxic effect when applied to porous substrates.

The anopheline mosquito mortality rates observed after 24 hours in huts sprayed with Actellic 300CS fell below the WHO threshold from the sixth month post-spraying in cement and mud huts, from the seventh month post-spraying in tin and wood huts, and from the eighth month post-spraying in vegetal huts. During the ninth month post-spraying, similarly to what was observed with SumiShield 50WG, mortality induced by Actellic 300CS within 24 hours fell below the WHO threshold in all treated huts. However, considering the mortality after 24 hours, Actellic 300CS is almost always more effective, regardless of the type of hut and post-spraying period. This result should be considered with caution because the number of mosquitoes exposed to Actellic 300CS was approximately four times lower than that of SumiShield 50WG for the same type of hut and the same post-spraying period. No comparison could be made when considering mortality after 48 hours or more. By killing wild anopheline mosquitoes that entered experimental huts built with different materials for eight months post-spraying, exhibiting a delayed killing effect on an insecticide-susceptible *Anopheles* strain for eight months, and achieving mortality rates exceeding the WHO threshold, SumiShield 50WG shows great potential for use in IRS programs in Madagascar.

The present study was designed as a proof-of-concept to assess the efficacy of SumiShield 50WG, a clothianidin-based formulation, for IRS programs targeting anopheline mosquitoes in Madagascar. SumiShield 50WG has since been adopted for IRS and is currently deployed in multiple districts across the country. The results of the current study support its continued use and suggest extending IRS coverage with SumiShield 50WG to additional districts, particularly those with prolonged malaria transmission seasons, given that no resistance to this insecticide has been reported to date.^19^
